# The Effect of Clinically Indicated Liraglutide on Pericoronary Adipose Tissue in Type 2 Diabetic Patients

**DOI:** 10.1155/2023/5126825

**Published:** 2023-01-14

**Authors:** Irmelin I. A. Biesenbach, Laurits J. Heinsen, Katrine S. Overgaard, Thomas R. Andersen, Søren Auscher, Kenneth Egstrup

**Affiliations:** ^1^Faculty of Health Science, University of Southern Denmark, Winsløwparken 19, 5000 Odense C, Denmark; ^2^Cardiovascular Research Unit, Odense University Hospital Svendborg, Baagøes Alle 15, 5700 Svendborg, Denmark

## Abstract

Vascular inflammation can be detected in the pericoronary adipose tissue (PCAT) by coronary computed tomography angiography (CCTA) attenuation. Treatment with liraglutide is associated with anti-inflammatory effects and reduces cardiovascular risk in diabetic patients. This study is aimed at examining the effect of clinically indicated liraglutide on PCAT attenuation. Asymptomatic patients with type 2 diabetes mellitus (T2DM) and without known ischemic heart disease underwent clinical examination, blood analysis, and CCTA. The main coronary arteries were outlined and PCAT attenuation was measured on the proximal 40 mm. Patients treated with liraglutide on a clinical indication were compared to patients not receiving liraglutide. The study included 190 patients; 53 (28%) received liraglutide (Lira+) and 137 (72%) did not (Lira-). There were no significant differences in PCAT attenuation between the two groups in either artery. However, PCAT attenuation measured around the left anterior descending artery (LAD) was lower in the Lira+ group after adjustment for age, sex, body mass index, and T2DM duration (*b* coefficient -2.4, *p* = 0.029). In a population of cardiac asymptomatic T2DM patients, treatment with clinically indicated liraglutide was not associated with differences in PCAT attenuation compared to nonliraglutide treatment in the unadjusted model. An association was seen in the adjusted model for the left anterior descending artery, possibly indicating an anti-inflammatory effect.

## 1. Introduction

Type 2 diabetes mellitus (T2DM) is one of the fastest growing health challenges worldwide [1]. It is estimated to contribute to 10% of deaths globally and almost half of these deaths occur in people under the age of 60 years [2]. The leading cause of morbidity and mortality is atherosclerotic cardiovascular disease (CVD) [3].

Vascular inflammation is a determining factor in CVD as it influences atherogenesis, plaque progression, and subsequent rupture [4–7]. Thus, vascular inflammation is an aggravating factor as well as a prognostic determinant for CVD [8]. Cytokines diffuse between the arterial wall and the adjacent pericoronary adipose tissue (PCAT) in a bidirectional paracrine manner [9]. Hyperglycemia and dyslipidemia lead to dysfunctional and inflamed PCAT, promoting the development of atherosclerosis [10]. The inflammation can be detected noninvasively with coronary computed tomography angiography (CCTA) attenuation [11]. In response to inflammation, the normal lipid-rich tissue (with Hounsfield unit (HU) values close to -190) changes in structure to a more aqueous form, resulting in less negative HU values (closer to -30). [12]. PCAT attenuation measured around the proximal segments of either one or all three major coronary arteries is associated with coronary artery disease presence and stage [12, 13], plaque burden [12, 14, 15], and cardiac and all-cause mortality [16]. PCAT attenuation measured around the proximal right coronary artery (RCA) is significantly higher in diabetic patients than in nondiabetic patients [17], suggesting a higher degree of inflammation in diabetic patients.

Liraglutide is a long-acting glucagon-like-peptide-1 (GLP-1) receptor agonist used in treatment of T2DM [18]. It lowers blood glucose by stimulating glucose-dependent insulin secretion and reducing glucagon secretion [19]. Moreover, it reduces body weight and systolic blood pressure [18]. In the LEADER trial, a randomized controlled study on patients with T2DM and high cardiovascular risk, treatment with liraglutide versus placebo added to standard care resulted in lower occurrence of major cardiovascular events [20]. The underlying mechanisms behind this beneficial effect of liraglutide are not fully understood. Recent findings have demonstrated that both cardiac adipose tissue [21] and macrophages [22] express GLP-1 receptors, suggesting a more direct and possibly anti-inflammatory effect of liraglutide on PCAT, besides the systemic antidiabetic effect. Liraglutide has shown anti-inflammatory properties in rats [23–26], cultured human endothelial cells [27–29], and in a randomized controlled trial [30]. On the other hand, a randomized, placebo-controlled trial could not report a reduction of arterial inflammation measured with [^18^F]fluorodeoxyglucose positron emission tomography/computed tomography after 26 weeks of liraglutide treatment [31]. The effect of liraglutide on PCAT inflammation quantified by CCTA attenuation has not yet been examined.

The aim of this study was to investigate the effect of clinically indicated liraglutide on pericoronary adipose tissue (PCAT) attenuation derived from CCTA, as a surrogate marker of pericoronary inflammation, in diabetic patients. This was an observational cross-sectional study of cardiac asymptomatic patients with T2DM, comparing PCAT attenuation values from patients receiving liraglutide to patients not receiving liraglutide, adjusted for known atherosclerotic risk factors.

## 2. Materials and Methods

Data for the current study was derived from a previous prospective observational single-center study performed at Odense University Hospital, Svendborg, Denmark, from March 2016 to September 2018 [32, 33]. The primary aim of the original study was to investigate the changes in plaque burden and composition in T2DM. The study was approved by the Regional Ethics Committee (ID S-20150029) as well as the Danish Data Protection agency (ID. 2008-58-0035).

### 2.1. Study Population

Patients included in the original study underwent clinical examination, blood analysis, and CCTA at baseline and at 1-year follow-up. Only baseline data were used in the current study and patient data were anonymized before entering the study. The population consisted of patients with T2DM, no history of ischemic heart disease (IHD), no previous percutaneous cardiac intervention or coronary artery bypass graft, and no clinical indication for CCTA. Patients were recruited through the Endocrinology Outpatient Clinic and the Retina Photography Clinic at Odense University Hospital, Svendborg, Denmark, from March 2016 to September 2017. Inclusion criteria were age ≥18 years, written informed consent, and a preexisting diagnosis of T2DM, diagnosed according to guidelines [34]. Exclusion criteria were: (1) history of IHD, presence of angina pectoris, and documented heart failure, (2) estimated glomerular filtration rate (eGFR) <45 mL/min/1.73m^2^, 3) allergy to iodine contrast, and (4) noninterpretable CCTA. For the current study, the following additional exclusion criteria were applied: (5) treatment with dipeptidyl peptidase 4 (DDP-4) inhibitors [20], (6) treatment with other GLP-1 receptor agonists, and (7) liraglutide treatment duration less than 12 months. Liraglutide was prescribed by physicians independent of this study and it was given on clinical indication in accordance with the Danish treatment guidelines [35].

### 2.2. Data Collection

Baseline characteristics such as age, sex, height, weight, body mass index (BMI), duration of diabetes, and smoking exposure in pack years were collected at the initial patient visit. Current medication intake, dosage, and duration were also collected in the initial patient visit and validated through online registers. Blood samples were drawn to analyze hemoglobin A1c (HbA1c), low-density lipoprotein cholesterol (LDL), high-density lipoprotein cholesterol (HDL), triglycerides, estimated glomerular filtration (eGRF), and high-sensitivity C-reactive protein (hsCRP), leukocytes and neutrophils. Patients were categorized as having hypertension if (1) systolic blood pressure ≥130 mmHg was measured on two separate occasions, (2) diastolic blood pressure ≥80 mmHg was measured on two separate occasions [36], or (3) patients received one or more antihypertensive drugs. Patients were categorized as having dyslipidemia if (1) LDL cholesterol >100 mg/dL (>2.6 mmol/L), (2) HDL cholesterol <50 mg/dL (<1.3 mmol/L), (3) triglycerides >150 mg/dL (>1.7 mmol/L) [36], or (4) if patients received one or more lipid-lowering drugs.

### 2.3. CCTA Acquisition

Pretreatment with 7.5 mg ivabradine daily was given two days prior to the scan to lower the heart rate [37]. On the day of the scan, intravenous beta-blocker was given to patients with heart rate >65 beats/min to further reduce heart rate. Sublingual fast-acting nitrate was administered immediately before the enhanced scan to maximally dilate the coronary arteries [38]. Images were obtained by a 256-detector system (GE revolution CT, Waukesha, Wisconsin, USA) by an electrocardiogram-gated prospective acquisition in the 75% of the R-R interval with additional padding of 45 ms to allow additional reconstruction. In patients with heart rate >65 beats/min, an additional phase was acquired in the 40% phase of the R-R interval. A repeated scan was acquired if the heart rhythm was irregular. 60 mL iodine contrast (Visipaque 320 mg iodine/mL) was injected at 5 mL/s, and the scan was performed when maximal attenuation was detected in the ascending aorta. Tube voltage and current were modulated to patient's BMI with a tube voltage between 80 and 140 kV and a tube current between 150 and 700 mA. Gantry rotation time was 280 ms with 16 cm axial coverage. The median radiation dose per scan was 2.01 millisievert. The slice thickness was 0.625 mm, and 40% adaptive statistical iterative reconstruction was adopted. All available phases were reconstructed, and images with superior image quality were selected for analysis.

### 2.4. PCAT Analysis

In the current study, CCTA-scans were analyzed on an offline workstation with validated semiautomatic software (QAngio CT Research Edition version 3.2.0.12, Medis, Leiden, NL). Centerlines of each coronary artery were automatically extracted by the software. Based on longitudinal images, cross-sectional lumen and vessel contours were created and manually corrected if necessary. PCAT was defined as the adipose tissue (HU ranging from -190 to -30) within a radial distance from the outer vessel wall equal to the diameter of the coronary vessel [12]. PCAT analysis of the right coronary artery (RCA) involved the proximal 10 mm to 50 mm of the vessel, excluding the first 10 mm to prevent noise from the aortic wall. The proximal 40 mm of the left anterior descending artery (LAD) and left circumflex artery (CX) were analyzed, excluding the left main coronary artery due to its variable length and possible absence [16]. All PCAT attenuation analysis was made by a single experienced observer blinded to liraglutide treatment. PCAT data were calculated as a mean by the QAngio software (Figure S1, Supplementary Material).

### 2.5. Statistical Analysis

Continuous data are presented as mean ± standard deviation (SD), categorical data are presented in percentages, and ordinal data are presented as median and interquartile range (IQR). Student's *t*-test for independent groups was used to compare continuous variables, and the chi-squared test was used for categorical variables. Quantile-quantile (qq) plots and histograms were used to assess whether PCAT data were normally distributed. To compare PCAT attenuation values between Lira+ and Lira-, Student's *t*-test for independent groups was applied. Multivariable linear regression was used to adjust for the following known atherosclerotic risk factors: age, sex, BMI, hypertension, dyslipidemia, and smoking. These variables were selected *a priori* based on literature review [39, 40]. T2DM duration was chosen *a posteriori* since the duration differed significantly between the groups (Table 1) and since T2DM duration has shown to be a predictor of coronary artery disease [41]. The adjustments are presented stepwise for transparency. A two-sided *p* value < 0.05 was considered statistically significant. All statistical analysis were performed using Stata (version BE 17.0, StataCorp, Texas, USA).

## 3. Results

**Figure 1:**
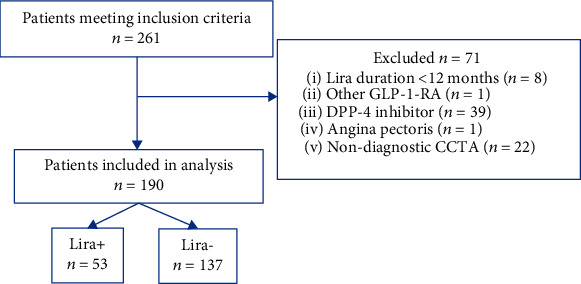
Study flow chart depicting recruitment, inclusion, exclusion, and clinically allocated treatment groups.

Two hundred and sixty-one patients with T2DM were eligible for participation, of whom 71 patients were excluded according to the exclusion criteria (Figure 1). Consequently, 190 patients were included for analysis, of whom 53 (28%) were treated with clinically indicated liraglutide and 137 (72%) did not receive liraglutide treatment.

### 3.1. Patient Characteristics

The mean age was 62 ± 10 years and 140 (74%) of the study patients were male. The comparison of baseline characteristics between Lira+ and Lira- are shown in Table 1. The Lira+ group was characterized by longer duration of diabetes, higher BMI, higher levels of HbA1c and triglycerides, a larger proportion of metformin and sulfonylurea users, and longer duration of lipid-lowering medication compared to the Lira- group. There was no difference in inflammatory markers (hsCRP, leukocytes, and neutrophils) between the two groups.

### 3.2. PCAT Attenuation

Coronary artery segments with insufficient image quality were excluded from the analysis. Consequently, analysis for CX included 182 (50 Lira+/132 Lira-) coronary segments, LAD included 188 (53 Lira+/135 Lira-), and RCA included 184 (52 Lira+/132 Lira-) coronary segments. Unadjusted differences in PCAT attenuation between the Lira+ and the Lira- group did not reveal any statistical significance for either coronary artery (Table 2).

In multivariable linear regression analysis, liraglutide treatment was significantly associated with lower PCAT attenuation values around the LAD when adjusted for age, sex, BMI, and T2DM duration (*b* coefficient -2.4, *p* = 0.029). The association remained significant after further adjustment for lipid-lowering medication, antihypertensive medication, and active smoking, as can be seen in Table 3. However, the significant association was tied to the T2DM duration and a *p* value < 0.05 could not be reached without this adjustment component. There was no significant difference in PCAT attenuation measured around RCA and CX between the two groups.

### 3.3. Plaque Burden

**Figure 2:**
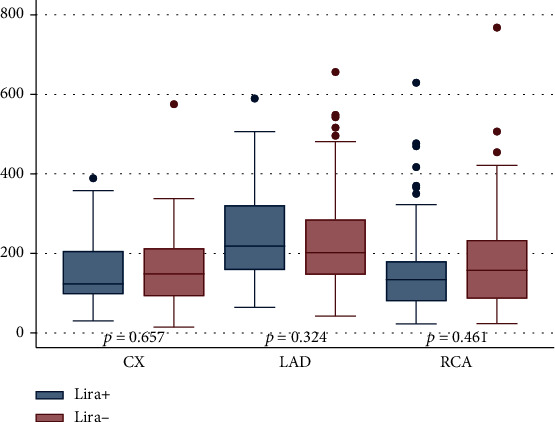
Plaque burden in the coronary arteries stratified by liraglutide treatment. Abbreviations: CX: left circumflex artery; LAD: left anterior descending artery; RCA: right coronary artery; *p* values were calculated using two-sample Wilcoxon's rank-sum (Mann–Whitney) test. Number of included segments: 182 CX, 188 LAD, and 184 RCA.

Since greater plaque burden is associated with higher PCAT attenuation values [12], we hypothesized that arteries with greater plaque burden could be more susceptible to the anti-inflammatory effects of liraglutide and provide a stronger anti-inflammatory signal compared to arteries with less plaque burden. Therefore, an explorative analysis of plaque burden in the three coronary arteries was performed. Plaque burden was defined as the total atheroma volume normalized by the segment length, calculated using the following equation: ((Vessel volume–Lumen volume)/Segment length)^∗^ Mean segment length in the population. Lumen and vessel contours were created automatically by the QAngio software and manually corrected if necessary. Analyses of plaque burden were made on the same 40 mm segments used for PCAT analyses. Plaque burden was significantly higher in the LAD compared to CX and RCA (Figure S2, Supplementary Material). There was no statistical difference between plaque burden in CX and RCA. Moreover, we analyzed the differences in plaque burden between Lira+ and Lira-. There were no statistical differences in plaque burden in the coronary arteries when stratified by liraglutide treatment (Figure 2).

## 4. Discussion

To the best of our knowledge, the present study is the first to examine how liraglutide treatment influences pericoronary adipose tissue (PCAT) using CTTA-derived attenuation as a surrogate marker of inflammation. Unadjusted differences in PCAT attenuation between the Lira+ and the Lira- group did not reveal any statistical significance for either coronary artery in a population of cardiac asymptomatic patients with type 2 diabetes mellitus (T2DM). However, in multivariable linear regression analysis, liraglutide treatment was significantly associated with lower PCAT attenuation values around the left anterior descending artery (LAD) when adjusted for age, sex, BMI, and T2DM duration.

In the LEADER trial, liraglutide compared to placebo led to a lower rate of first occurrence of death from cardiovascular causes, nonfatal myocardial infarction or nonfatal stroke, and a lower rate of death from any cause in the time-to-event analysis [20]. We hypothesized that the cardiovascular risk reduction could in part be a result of anti-inflammatory properties of liraglutide. In the LIRAFLAME trial [31], treatment with liraglutide for 26 weeks did not change vascular inflammation assessed by [^18^F]fluorodeoxyglucose (FDG) uptake measured in the carotid arteries, thoracic aorta, and abdominal aorta compared with placebo. Both the current study and LIRAFLAME indicate that liraglutide treatment does not exhibit anti-inflammatory effects on human arteries. On the other hand, studies on nondiabetic [23] and type 2 diabetic [24–26] rats as well as studies on cultured human vascular endothelial cells [27–29] have disclosed that liraglutide exerts marked antioxidant and anti-inflammatory properties at therapeutic plasma concentrations [42]. Moreover, in a randomized controlled trial, liraglutide treatment increased plasma levels of the anti-inflammatory and antidiabetic adipokine adiponectin, which is ordinarily reduced in T2DM [43] compared to glimepiride treatment [44]. Therefore, it is possible that liraglutide has an anti-inflammatory effect on arteries, which both the current study and the LIRAFLAME [31] study could not measure.

In the LIRAFLAME [31] study, exploratory analyses found that patients with known cardiovascular disease (CVD) had a 10% higher carotid FDG uptake at baseline than patients without CVD. In a subgroup analysis of CVD patients, a significant reduction in FDG uptake was observed for liraglutide and not placebo. However, the difference between liraglutide and placebo did not reach statistical significance, perhaps due to the subgroup size (*n* = 23). These results could indicate that the reason both LIRAFLAME [31] and the current study (excluding all patients with ischemic heart disease, presence of angina pectoris, and documented heart failure) did not reveal any anti-inflammatory effect of liraglutide was because both studies were made on patients at low CVD risk. This low risk would result in lower arterial inflammation and in a reduced potential for decreasing the inflammatory signal compared to a high-risk population. Interestingly, a substudy of LIRAFLAME demonstrated a significant reduction in [^64^Cu]Cu-DOTATATE uptake in the coronary arteries in the liraglutide group and not in the placebo group [45], suggesting that this method could be more sensitive than PCAT attenuation and FDG uptake. [^64^Cu]Cu-DOTATATE is a radioactive tracer targeting activated macrophages in the vessel-wall used to quantify inflammation in the coronary arteries.

Sirtuin 6 (SIRT6) is a nuclear deacetylase that protects against vascular inflammation [46]. Not only GLP-1 receptor agonists [47] but also sodium glucose cotransport 2 inhibitors (SGLT2i) [48] and metformin [49] have shown to increase SIRT6 expression and exhibit anti-inflammatory effects on cardiac plaque. Moreover, GLP-1 receptor agonists [50], SGLT2i [51], and metformin [52] have shown to significantly reduce the rate of major adverse cardiovascular events in T2DM. In the current study, there was no significant difference in SGLT2i usage between the two groups. However, there were significantly more metformin users in the Lira+ group compared to Lira- (Table 1), which might have led to an overestimation of liraglutide's anti-inflammatory effect.

The current study found that liraglutide treatment was associated with significantly lower PCAT attenuation values around the LAD when adjusted for age, sex, BMI, and T2DM duration. The association remained significant after further adjustment but was dependent on the T2DM duration (Table 3). The decision to adjust for T2DM duration was chosen *a posteriori* since the duration differed significantly between the two groups (Table 1) and since T2DM duration has shown to be a predictor of coronary artery disease and of adverse cardiovascular events [41]. Moreover, the Lira+ group was characterized by higher BMI, higher levels of HbA1c and triglycerides, and longer duration of lipid-lowering medication compared to the Lira- group. It is our hypothesis that these traits characterizing the Lira+ group could have translated into more diseased coronary arteries with a higher degree of inflammation prior to liraglutide treatment and that this high degree of inflammation could then have been reduced by the liraglutide treatment ending up in the same level as the Lira- group (Table 1). It is therefore possible that the adjusted model, observing lower PCAT attenuation values (and thereby a lower degree of inflammation) around the LAD in the Lira+ group compared to the Lira- group, is more applicable than the unadjusted model.

In the CRISP-CT study, increased PCAT attenuation around LAD and RCA were predictive of all-cause and cardiac mortality in a population undergoing clinically indicated diagnostic CCTA [16]. It was uncertain whether these results could be applied to T2DM patients since they are at a considerably higher cardiovascular risk [53, 54] and have a higher degree of coronary inflammation [17] than nondiabetics. However, a recent study on T2DM patients undergoing clinically indicated diagnostic CCTA confirmed that high PCAT attenuation around LAD was significantly associated with cardiovascular events [55]. Interestingly, both the beforementioned study on high-risk T2DM patients and the present study on low-risk T2DM patients only found a significant association to LAD, and not RCA or CX.

Antonopoulos et al. found that PCAT attenuation was positively related to atherosclerotic plaque burden [12]. Coronary atherosclerosis demonstrates a predilection for certain parts of the coronary vessels. Particularly, the proximal LAD is the most common location for thin cap fibroatheromas and rupture plaque [56, 57]. This was also the case in the present study, and we found a higher plaque burden in the LAD compared to RCA and CX (Figure S2). This may suggest that the inflammatory effect of liraglutide is more pronounced in the vessel with the highest atherosclerotic burden.

Goeller et al. showed that PCAT attenuation dynamically followed changes in plaque burden with a significant decrease in PCAT attenuation in patients with decreased plaque burden and vice versa [15]. Due to this interaction, it is possible that if liraglutide decreases PCAT attenuation, it could furthermore lead to a reduction in plaque burden. However, in the present study, differences in plaque burden between Lira+ and Lira- were nonsignificant (Figure 2).

Since inflammation is not only associated with plaque burden but also with plaque instability and rupture [4–7], it is plausible that liraglutide in the current study population could mainly have led to a shift from vulnerable plaques to more stable plaque types.

### 4.1. Strengths and Limitations

The major limitation of this study is the cross-sectional design, with lack of randomization, blinding and a placebo-controlled group. This led to a selection of patients in the liraglutide group who were in poorer health and with more cardiovascular risk factors, which could have translated into more diseased coronary arteries compared to the patients who did not receive liraglutide. This observational design was performed with a hypothesis generating purpose. The findings of the study warrant further prospective studies to confirm the results. A randomized controlled study with a placebo-control would reduce possible bias and more reliably establish a cause-effect relationship between the liraglutide intervention and PCAT attenuation outcome. Moreover, this study had a relatively limited sample size.Last, in the current study, mean CT attenuation was used for analysis, whereas the CRISP-CT study used the Fat Attenuation Index (FAI). The FAI adjusts for obesity status, technical scanning parameters, and anatomical factors [11], but because the algorithm was nondisclosed by the authors, it is unclear how these adjustments were made.

### 4.2. Perspectives

Recently, the CANTOS [6] and COLCOT [58] trials provided clinical evidence that targeting systemic inflammation which improves cardiovascular disease outcome. Thus, making it conceivable that the cardiovascular protective effects of liraglutide could in part stem from its anti-inflammatory properties. Further studies are needed to confirm this. Moreover, prospective randomized controlled studies are needed to evaluate the effect of liraglutide on plaque burden, plaque composition, and plaque stabilization. Hopefully, broader implementation of antidiabetic and anti-inflammatory treatment could reduce morbidity and mortality in the fast-growing population suffering from T2DM.

## 5. Conclusion

In a population of cardiovascular asymptomatic patients with type 2 diabetes, treatment with clinically indicated liraglutide was not associated with differences in pericoronary adipose tissue (PCAT) CCTA attenuation compared to no liraglutide treatment in the unadjusted model. However, in the adjusted model, liraglutide was associated with lower PCAT attenuation values measured around the left anterior descending artery compared to no liraglutide treatment. Our findings might indicate that the cardioprotective effect of liraglutide could in part come from anti-inflammatory properties, dampening inflammation in the coronary arteries, and possibly reducing atherogenesis and plaque vulnerability.

## Figures and Tables

**Table 1:** Baseline characteristics of T2DM patients, total and stratified by liraglutide treatment.

	Total *n* = 190	Lira+ *n* = 53	Lira- *n* = 137	*p* value
Age (years) mean ± SD	61.8 ± 9.6	61.6 ± 9.2	61.9 ± 9.8	0.835
Male, *n* (%)	140 (74)	36 (68)	104 (76)	0.262
T2DM duration (years), mean ± SD	10.6 ± 7.2	13.4 ± 6.8	9.5 ± 7.0	**<0.001**
BMI (kg/m^2^), mean ± SD	30.7 ± 4.9	32.3 ± 5.0	30.1 ± 4.7	**0.004**
Hypertension, *n* (%)	185 (97)	53 (100)	132 (96)	0.159
Dyslipidemia, *n* (%)	183 (96)	52 (98)	131 (96)	0.413
Active smoker, *n* (%)	71 (37)	25 (47)	46 (34)	0.082
Pack years (years/20 cigarettes daily), mean ± SD	15.2 ± 19.1	13.1 ± 16.3	16.1 ± 20.1	0.340
Tube voltage (kV), median (IQR)	120 (100-140)	120 (100-120)	100 (100-140)	0.271
*Medication*				
Insulin, *n* (%)	82 (43)	25 (47)	57 (42)	0.487
Metformin, *n* (%)	158 (83)	50 (94)	108 (79)	**0.010**
Sulfonylurea, *n* (%)	30 (16)	14 (26)	16 (12)	**0.012**
SGLT2 inhibitor, *n* (%)	18 (9)	8 (15)	10 (7)	0.100
Lipid-lowering, *n* (%)	152 (80)	47 (89)	105 (77)	0.063
Lipid-lowering duration (years), mean ± SD	7.4 ± 5.6	9.0 ± 4.4	6.7 ± 5.9	**0.016**
Antihypertensive, *n* (%)	138 (73)	39 (74)	99 (72)	0.855
*Laboratory findings*				
HbA1c (mmol/L), mean ± SD	60.0 ± 13.9	65.5 ± 15.5	57.8 ± 12.7	**<0.001**
Total cholesterol (mmol/L), mean ± SD	4.0 ± 0.9	4.0 ± 0.9	4.1 ± 0.9	0.408
LDL (mmol/L), mean ± SD	2.0 ± 0.8	1.9 ± 0.8	2.0 ± 0.8	0.463
HDL (mmol/L), mean ± SD	1.2 ± 0.6	1.2 ± 0.3	1.3 ± 0.7	0.472
Triglycerides (mmol/L), mean ± SD	2.1 ± 1.4	2.5 ± 1.9	1.9 ± 1.1	**0.006**
*Inflammatory markers*				
hsCRP (mg/L), median (IQR)	1.4 (0.6-3.4)	1.3 (0.6-3.6)	1.4 (0.6-3.2)	0.992
Leukocytes, mean ± SD	7.01 ± 1.93	7.01 ± 1.86	7.10 ± 1.96	0.948
Neutrophils, mean ± SD	4.43 ± 1.52	4.38 ± 1.45	4.44 ± 1.55	0.785

Abbreviations: T2DM: type 2 diabetes mellitus; Lira+: in clinically indicated liraglutide treatment; Lira-: no liraglutide treatment; SD: standard deviation; *n*: number; IQR: interquartile range; BMI: body mass index; SGLT2: sodium glucose cotransport 2; HbA1c: hemoglobin A1c; LDL: low density lipoprotein cholesterol; HDL: high density lipoprotein cholesterol; hsCRP: high sensitivity C-reactive protein. Patients were categorized as having hypertension if a blood pressure ≥130/80 mmHg was measured on two separate occasions or if patients received antihypertensive drugs. Patients were categorized as having dyslipidemia if (1) LDL cholesterol >2.6 mmol/L, (2) HDL cholesterol <1.3 mmol/L, (3) triglycerides >1.7 mmol/L, or (4) if patients received lipid-lowering drugs.

**Table 2:** Differences in PCAT CCTA attenuation crude values between Lira+ and Lira- for each coronary artery.

Unadjusted	Lira+	Lira-	*p* value
CX (mean ± SD) HU	−74.42 ± 6.69	−74.61 ± 6.07	0.859
LAD (mean ± SD) HU	−82.43 ± 5.69	−80.98 ± 6.97	0.179
RCA (mean ± SD) HU	−80.03 ± 6.90	−79.91 ± 7.43	0.926

Abbreviations: CX: left circumflex artery; LAD: left anterior descending artery; RCA: right coronary artery; Lira+: in clinically indicated liraglutide treatment; Lira-: no liraglutide treatment; SD: standard deviation; HU: Hounsfield unit. Coronary artery segments with insufficient image quality were excluded from the analysis. Consequently, analysis for CX included 182 (50 Lira+/132 Lira-), LAD included 188 (53 Lira+/135 Lira-), and RCA included 184 (52 Lira+/132 Lira-) segments.

**Table 3:** Multiple linear regression: unadjusted and adjusted differences in PCAT attenuation between Lira+ and Lira- for each coronary artery.

	CX		LAD		RCA	
	*b* coefficient (95% CI)	*p* value	*b* coefficient (95% CI)	*p* value	*b* coefficient (95% CI)	*p* value
Unadjusted	0.185 (-1.86; 2.23)	0.859	-1.452 (-3.58; 0.67)	0.179	-0.110 (-2.46; 2.24)	0.926
Adjusted for age, sex, and BMI	-0.063 (-2.04; 1.92)	0.950	-1.645 (-3.75; 0.45)	0.124	0.15 (-2.18; 2.48)	0.898
Adjusted for age, sex, BMI, and T2DM duration	-0.242 (-2.29; 1.81)	0.816	-2.417 (-4.58; -0.25)	**0.029**	-0.149 (-2.61; 2.31)	0.905
Adjusted age, sex, BMI, lipid-lowering medication, antihypertensive medication, and active smoking	-0.060 (-2.07; 1.95)	0.953	-1.907 (-4.02; 0.21)	0.077	0.499 (-1.85; 2.85)	0.676
Adjusted for age, sex, BMI, lipid-lowering medication, antihypertensive medication, active smoking, and T2DM duration	-0.291 (-2.36; 1.78)	0.782	-2.607 (-4.78; -0.44)	**0.019**	-0.078 (-2.53; 2.38)	0.950

Abbreviations: CX: left circumflex artery; LAD: left anterior descending artery; RCA: right coronary artery; Lira+: in clinically indicated liraglutide treatment; Lira-: no liraglutide treatment; BMI: body mass index; T2DM: type 2 diabetes mellitus; CI: confidence interval; HU: Hounsfield unit. Coronary artery segments with insufficient image quality were excluded from the analysis. Consequently, analysis for CX included 182 (50 Lira+/132 Lira-), LAD included 188 (53 Lira+/135 Lira-), and RCA included 184 (52 Lira+/132 Lira-) segments. The *b* coefficients and the 95% CI are for liraglutide in the multiple linear regression models.

## Data Availability

The observational data used to support the findings of this study were supplied by the Cardiovascular Research Unit, Odense University Hospital Svendborg, under license and so cannot be made freely available. Requests for access to these data should be made to Kenneth Egstrup, kenneth.egstrup@rsyd.dk.

## Supplementary Materials

Supplementary Materials S1: figure illustrating the pericoronary adipose tissue attenuation in the QAngio software. S2: box plot depicting differences in plaque burden in the coronary arteries in the whole study population.
