# Supplementary material for: The Effect of Clinically Indicated Liraglutide on Pericoronary Adipose Tissue in Type 2 Diabetic Patients
Source: Cardiovasc Ther. 2023 Jan 14;2023:5126825. doi: 10.1155/2023/5126825 (PMC9867582; doi:10.1155/2023/5126825)
Supplement: Supplementary Materials — S1: figure illustrating the pericoronary adipose tissue attenuation in the QAngio software. S2: box plot depicting differences in plaque burden in the coronary arteries in the whole study population. [file 5126825.f1.docx]

# Supplementary Material

**Figure S1:** Coronary artery with illuminated pericoronary adipose tissue between − 190 and – 30 HU in the longitudinal, curved view A) and in the cross-sectional view B).


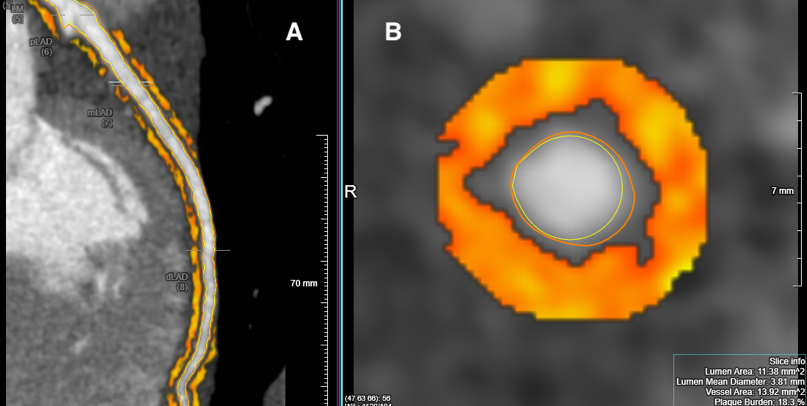


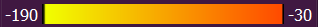


**Figure S2:** Plaque burden in the coronary arteries.

Abbreviations: CX: left circumflex artery, LAD: left anterior descending artery, RCA:
right coronary artery, Means and *p-*values were calculated using all available segments;
182 CX, 188 LAD and 184 RCA. *p-*values were calculated using Wilcoxon signed-rank test.
